# Is It Possible to Apply Agile Methods to Contribute to the Linux Kernel?

**DOI:** 10.1007/978-3-030-58858-8_30

**Published:** 2020-08-18

**Authors:** Thatiane de Oliveira Rosa, Alfredo Goldman

**Affiliations:** 6 IT University of Copenhagen, Copenhagen, Denmark; 7 University of British Columbia, Vancouver, BC Canada; 8 University of São Paulo, São Paulo, SP Brazil; 9 Federal Institute of Tocantins, Paraíso do Tocantins, TO Brazil

**Keywords:** Agile methods, Linux kernel, Low-level programming, XP Lab, Teaching challenges

## Abstract

In this document, we describe the experience of teaching Agile Methods for developing projects related to the Linux Kernel, during the XP Lab course. In 2018, the first project related to this context emerged. This project had the objective of making adjustments to the driver for Linux IIO subsystem. The second project was developed in 2019 and aimed to refactor the Ethernet driver used in the kernel of a Brazilian Single Board Computer. Based on 19 years of experience offering the XP Lab course, we consider the development of these projects to be a challenging teaching activity, which deserves to be presented and discussed with students, educators, and professionals. Our aim is to show that it is possible to adapt Agile Values to different software development settings.

## Introduction

There are several challenges related to the teaching-learning process of Agile Methods in the academic context. The ideal is that students learn the theoretical concepts and have a practical experience close to the industry reality. Since 2001, the Institute of Mathematics and Statistics of the University of São Paulo annually offers the eXtreme Programming Laboratory (XP Lab) course, which aims to teach Agile Methods in practice, where students deal with real customers and projects and follow the XP values and principles [4]. From the offer of this course, we can deal with different teaching challenges, contexts, and development projects, as well as to evolve and continuously improve.

Recently, we were faced with a new challenge: to teach the adoption of Agile Methods to develop projects that deal with low-level programming. In 2018, we had a project in our Agile Methods course related to the Linux Kernel IIO Staging Drivers. In 2019, we had another project that the goal was to refactor the Ethernet driver of a new device. In this paper, we describe how we adapted our mindset to do Agile in these particular software development environments.

The rest of this experience report is organized as follows. Section 2 presents our XP Lab course. Sections 3 and 4 describe the two projects developed during 2018 and 2019 related to low-level programming. Section 5 discusses how we adapted to support the development and present some lessons learned. The last section presents some final thoughts and remarks.

## XP Lab Course

XP Lab (eXtreme Programming Laboratory) is a course offered annually by IME/USP (Institute of Mathematics and Statistics of University of São Paulo) since 2001 for undergraduate and graduate students in Computer Science. The main goal is to teach Agile Methods in practice, providing the student with real knowledge and experience [3]. To achieve this goal, during the course, students develop real projects with real customers. The projects are developed by the students following the XP (eXtreme Programming) values - communication, simplicity, feedback, respect, and courage - and principles such as continuous improvement, incremental changes, and mutual benefit [1].

Furthermore, students use the original XP practices such as pair programming, shared code, stand-up meetings, and simple design, and other practices such as whole-class retrospectives in fishbowl format, lightning talks at lunchtime, rotation of team members across teams, brainwriting, coding dojos, test day, and refactoring day. During the first classes and in a punctual and opportune manner during the course, lectures and practical activities are held on relevant subjects such as Agile Methods and practices, DevOps, software architecture and patterns, continuous integration, technological stack, software testing, and technical debt. During the course, it is possible to notice that learning evolved, and students started to continuously learn and share technical knowledge, projects, Agile Methods, and skills [3].

Since 2001, over 500 students have been taught to adopt Agile Methods, 89 projects were executed, and over 10 companies attended [4]. During 19 years of offering, we had to adapt to several different contexts and situations.

In the last two years, we have faced the challenge of developing projects to contribute to the Linux Kernel. In the two next sections, we present an overview of these projects, outline the development process, some practices used, challenges faced, outcomes, and the evaluation process adopted.

## IIO Staging Drivers Project

This project was proposed by Rodrigo Siqueira^1^, an autonomous Linux Kernel contributor. The purpose was to create a development and contribution cycle to the Linux Kernel, disseminating the desired procedures to collaborate with the Linux Kernel’s evolution, in partnership with Kernel maintainers in Brazil. The main planned tasks were: basic contributions such as preparation of the development environment, aiming the code style adjustments; intermediary contributions such as minor bug fixes and refactorings; and advanced contributions, such as adjustments to the driver for the IIO subsystem [2].

Since XP Lab students did not know about Linux Kernel development, it was proposed contributing to staging drivers (drivers that need more adjustment, before being added to the live Kernel tree). Therefore, during the XP Lab course, students adopted Agile Development Methods to improve Linux test drivers. Furthermore, adjusted drivers should meet quality requirements to be appropriately added to the Linux Kernel.

The team of this project was formed by six students and two external coaches (Linux Kernel contributors). The customer was the Linux Kernel Community. During the project development, different face-to-face meetings with the customer were held to discuss and plan tasks. At the beginning of each class (twice a week), a stand-up meeting was held. Once a week, a member selected by the team participated in a stand-up meeting with the coaches of other projects that were being developed in the course. The purpose of this meeting was to share experiences among the teams. The team held regular meetings with the customer to discuss and plan tasks and priorities. Furthermore, at the end of each sprint, the team held a retrospective meeting. One of the most useful and used Agile Practices was pair programming.

As reported by the team members, the main challenge faced during the project development was the distance or unavailability of customers. The strategy adopted to mitigate this challenge was reading the available documentation to minimize the impacts of the lack of interaction with the customer.

At the end of the project, the team members reported that they could learn fundamental concepts of FLOSS (Free/Libre and Open Source Software) development. The main concepts learned were: the importance of clear and complete documentation and the importance of searching information in this documentation; recognize the importance of code reviews; use of tools that facilitate software development for Linux; workflow and development process in the Linux Kernel; and importance of sharing the code with the community. Furthermore, the team emphasizes that communication between developers, specialists (external coaches), and the community is essential for successful FLOSS development.

Another relevant outcome is that during the course, the team accounted for 20% of the contributions to the Linux IIO Subsystem. Furthermore, at the end of the course, the team members founded an extension group called FLUSP^2^, which aims to contribute to FLOSS projects. Among their main achievements we have: FLUSP was one of the top contributors for the kernel drivers for a while; one of our former students is now contributing to GCC as a committer; one of our former students was responsible for parallelizing the grep command of GIT. More details and information about this project is available in the following GitHub repositories: github.com/rodrigosiqueira/kworkflow and gitlab.com/groups/kernel-usp/.

## Labrador Project

The Labrador is a Brazilian Single Board Computer (SBC) developed by Caninos Loucos that works with open hardware and software. Caninos Loucos is an organization that develops SBCs with an open structure (hardware and software) for the Internet of Things (IoT). It is an initiative of the Technological Integrated Systems Laboratory (LSI-TEC) with the support of Polytechnic School of the University of São Paulo (Poli-USP) and Jon “Maddog” Hall, Board Director of the Linux Professional Institute [5].

The proposed project for the XP Lab course was to adopt Agile Development Methods to refactor the Ethernet driver used in the Labrador Kernel. This driver used obsolete Linux Kernel functions and must be updated. Such updates were necessary to enable the wide distribution of Labrador SBC.

The team of this project was formed by two students and three external coaches (domain specialists). The customers were the Caninos Loucos members. The development process and practices adopted in this project were very similar to the IIO Staging Drivers Project. At the beginning of each class (twice a week), a stand-up meeting was held. Once a week, the two members participated in a stand-up meeting with the coaches of other projects that were being developed in the course. The team held regular face-to-face meetings with the customer to discuss and plan tasks and priorities. Furthermore, at the end of each sprint, the team held a retrospective meeting.

According to the team, the main challenges faced are related to communication with the customers and the complexity of the legacy architecture and code. Communication with the customer was unsatisfactory, and it was difficult to get constant feedback. Given the complexity of the legacy architecture and code, it was difficult to define objectives and dimension the sprints. This last problem hindered the measurement of the progress of the driver refactoring work. Furthermore, given the difficulties listed, students were unable to adopt strategies to automate tests or carry out effectively continuous integration.

In order to mitigate the communication problem, the team sought other information sources. Thus, the students tried to use discussion forums and sought members of the Labrador project with higher availability. Regarding the complexity of the legacy architecture and code, the team readjusted expectations and broke the work into smaller issues.

At the end of the project, the team members reported that they could learn about the importance of efficient communication, constant feedback, clear and complete documentation, and proper software architecture.

The main results of this project include the refactoring of a part of the Labrador Ethernet driver and redefining the used software architecture. Furthermore, students resolved a Linux Kernel bug (approved patch), which influenced the Labrador driver’s refactoring. More details and information about this project is available in the following GitHub repository: github.com/r0zbot/labrador-linux.

## Lessons Learned

In the XP Lab course, the students are assessed continuously and incrementally. The final grade is defined based on the analysis of the following elements: Class attendance; Compliance with four extra hours per week; Self-evaluation; The internal coach’s evaluation of each team member (coach is a team member with greater knowledge of Agile Methods); Team evaluation by the customer; Team evaluation by meta-coach (graduate student with deep knowledge of Agile Methods, who supports the professor and guides the students).

The team evaluation by meta-coach is divided into three stages, where different requirements are analyzed. During stage 1, it is analyzed if the infrastructure has been installed and if the team is organized. In stage 2, the meta-coach analyzes the internal and external communication of each team and observes the activities’ planning and recording. Furthermore, the meta-coach monitors the rotation of pairs, the use of repositories, the realization of commits and tests, and the initialization of the continuous integration practice. In the last evaluation stage, elements such as tracking documentation, continuous integration, implementation of test-driven development, test coverage index, deliveries made to the customer, self-organization of the team during the course, and publication of “artifacts” to ensure the project continuity are considered.

However, when trying to adopt the same criteria for evaluating projects related to the Linux Kernel, we realized some could be adopted, and others could not. Among the inadequate criteria, we can mention continuous integration, test-driven development, test coverage, and deliveries. It is because, considering the development close to the hardware, it is very complex to create a continuous integration pipeline or obtain a good test coverage index. Therefore, it was necessary to adjust the criteria evaluated by the meta-coach.

To adapt the evaluation process, we return to the origin of Agile Methods; that is, we consider adherence to the values and principles of the Agile Manifesto [6]. For that, we monitored the teams’ work closely and made a checklist, composed by items such as customer satisfaction through valuable software, team adaptation to late changes, working software after a few weeks of development, team members engagement, face-to-face communication, simplicity of design, and self-organize ability. In the end, we were surprised at how the Agile Values were present.

We believe that the main lessons learned from the development of the projects presented were:

- Is recommended that the team has at least one coach (internal or external) who is a member of the FLOSS community and knows the Linux Kernel workflow and development process;
- It is fundamental to adopt the original Values and Principles of the Agile Manifesto to assist in the evaluation of projects developed in agile contexts.

## Final Remarks

The main objective of this document was to share the experience of adopting Agile Methods to develop software that deals with low-level programming. This type of project is especially challenging because it is not trivial to combine the teaching of Agile Methods with development close to the hardware.

We consider that our experience was successful, and we could provide relevant contributions to educators and the Agile and Free Software Communities. Furthermore, we were quite happy to leave our comfort zone and see that it is possible to apply Agile Principles and Methods to different environments. Therefore, the main conclusion we reached with the development of this work was to realize the simplicity, relevance, versatility, and transversality of the Agile Values and Principles.

We hope to have the opportunity to adopt and teach Agile Methods in new projects that deal with low-level programming and in other challenging contexts.

## Footnotes

1 https://www.linkedin.com/in/rodrigosiqueirajordao/.

2 https://flusp.ime.usp.br/.
